# Gut Microbial Composition and Antibiotic Resistance Profiles in Dairy Calves with Diarrhea

**DOI:** 10.3390/life15010010

**Published:** 2024-12-26

**Authors:** Lu Zhang, Jun Bai, Qian Guo, Long Li, Yanqing Jia, Xinxin Qiu, Dong Zhou, Zhencang Zhang, Huafeng Niu

**Affiliations:** 1 Department of Animal Engineering, Yangling Vocational & Technical College, Yangling 712100, China; luzhang2021@nwafu.edu.cn (L.Z.); wshbj@163.com (J.B.); lilong1101@126.com (L.L.); yqjia1987@163.com (Y.J.); ylzyqiuxinxin@163.com (X.Q.); 2 Shaanxi Engineering Research Center of the Prevention and Control for Animal Disease, Yangling Vocational & Technical College, Yangling 712100, China; 3 Key Laboratory for Efficient Ruminant Breeding Technology of Higher Education Institutions in Shanxi Province, Yangling Vocational and Technical College, Yangling 712100, China; 4 The Youth Innovation Team of Shaanxi Universities, Yangling Vocational and Technical College, Yangling 712100, China; 5 ShaanXi Province Management Station of Animal Health and Slaughter, Xi’an 710000, China; 18392957099@163.com; 6 College of Veterinary Medicine, Northwest A&F University, Yangling 712100, China; zhoudong1949@nwafu.edu.cn

**Keywords:** calf diarrhea, microbiota, high-throughput analysis, resistant genes

## Abstract

Calf diarrhea is a prevalent and significant health issue in dairy farming, severely impacting feed intake, weight gain, and survival rates in young calves. This study aimed to investigate the microbial composition and antibiotic resistance profiles of diarrheic calves to provide insights into the epidemiology and management of the condition. The prevalence of diarrhea in 1685 calves was analyzed. Rectal fecal samples were collected from five healthy and five diarrheic Holstein calves on a large dairy farm in Shaanxi Province, China. High-throughput 16S-rRNA sequencing and PCR were utilized for microbial and resistance gene analysis. In 2023, the overall diarrhea rate among 1685 calves was 9.08%, with a significantly higher diarrhea rate during the suckling period (8.13%) compared to the post-weaning period (0.95%) (*p* < 0.001). No differences in species diversity and richness were detected among the different groups. However, LEfSe analysis identified six genera (*Eubacterium, Eubacteriaceae, Prevotella, Comamonadaceae, Comamonas*, and *Firmicutes*) significantly enriched in diarrheic calves compared to healthy ones (LDA scores > 2, *p* < 0.05). Additionally, antibiotic resistance genes for quinolones, β-lactams, chloramphenicol, tetracyclines, and aminoglycosides were detected, with significantly higher prevalence in diarrheic calves. These findings demonstrate distinct microbial and antibiotic resistance profiles between healthy and diarrheic calves, emphasizing the importance of microbial management in controlling calf diarrhea.

## 1. Introduction

Diarrhea is a common and significant health issue in cattle, particularly prevalent among calves during the nursing period [1]. This condition poses a serious threat to calf health and leads to substantial economic losses for dairy farms [2]. Data from the U.S. Department of Agriculture indicate that, in 2013, 56.4% of all deaths among nursing heifers in the United States were attributed to diarrhea or other digestive tract diseases [3]. Similarly, a study of 1361 newborn calves in Korea found a total mortality rate of 10.7%, with deaths from digestive tract diseases, including diarrhea, accounting for 53.4% [4]. On large-scale Chinese farms, diseases among replacement calves are most prevalent during the nursing period, accounting for 51.4% of the total diseased cattle, with calf diarrhea comprising a striking 72.8%. These statistics underscore the urgent need for effective prevention and treatment strategies to mitigate the impact of calf diarrhea.

The pathogenesis of calf diarrhea is multifaceted, involving a combination of environmental, nutritional, immunological, and microbial factors [5]. Of these factors, a balanced intestinal microbiota is critical for maintaining calf health [6]. Numerous studies have demonstrated a strong association between the composition of the intestinal microbiota and the development of gastrointestinal diseases in mammals. [7,8,9]. Disruption of the intestinal microbial community can lead to impaired intestinal barrier function, increasing susceptibility to pathogens that can cause diarrhea [10,11]. Furthermore, harmful bacteria such as *Escherichia coli* and *Salmonella* may play significant roles in the onset of diarrhea [12]. Other pathogens, including viruses and parasites, can also contribute to diarrheal diseases in calves. For example, Bovine Viral Diarrhea Virus, Rotavirus, Torovirus, and Cryptosporidium are common infectious agents that affect the gastrointestinal tract and are often associated with calf diarrhea [13,14,15,16]. However, due to differences in feeding practices and environmental conditions, the structure of the intestinal microbiota and the types of pathogens responsible for diarrhea may vary across different farms. Investigating the relationship between calf microbiota structure and diarrhea in specific farming contexts is essential for devising targeted prevention and treatment strategies.

In addition, the extensive use of antibiotics in veterinary practice has led to cattle, including calves, becoming potential reservoirs for antibiotic resistance genes. Quinolones, which inhibit bacterial DNA synthesis, are represented by resistance genes such as *gyrA* and *gyrB*; β-lactams, essential for inhibiting bacterial cell wall synthesis, are marked by *blaTEM* and *blaSHV* genes; chloramphenicol resistance genes include *floR* and *catA1*; and tetracycline resistance is commonly associated with *tetB* and *tetD* [17,18]. Additionally, aminoglycoside resistance genes like *aadB* and *aadA1* are of interest due to their association with resistance to crucial antibiotics in this class.

In this study, we aimed to investigate the role of the gut microbiota in the progression of diarrhea in calves and to identify bacterial genera associated with diarrheal disease. Additionally, we analyzed antibiotic resistance genes to assess their potential prevalence in calves. The findings of this study may contribute to the development of effective strategies for disease prevention and intervention.

## 2. Materials and Methods

### 2.1. Calves Management and Fecal Sample Collection

A conventional cattle farm located in Shaanxi Province, China, was selected for the study, focusing on calves born on the ranch and monitored for health status before and after weaning in 2023. A total of 1685 calves were collected. This farm was chosen due to its representative large-scale intensive farming practices and well-documented calf management records. Holstein pre-weaning calves aged 60 ± 2 days were selected for microbiota analysis to ensure consistency in developmental stage and gut microbiota maturation. Calves treated with anthelmintics or antibiotics in the two months preceding sampling were excluded to minimize external influences on the microbiota.

For fecal sampling, strict aseptic techniques were employed. Fecal samples were collected from the rectal–anal junction (RAJ) using two sterile swabs to minimize contamination. A total of 10 samples were selected: 5 from calves with normal fecal samples (healthy group) and 5 from calves with diarrheal fecal samples (diarrheic group). Each sample was immediately transferred to 15 mL sterile centrifuge tubes and preserved in liquid nitrogen on-site. The samples were subsequently transported to the laboratory within 12 h under continuous cryogenic conditions for DNA extraction and further analysis.

### 2.2. DNA Extraction, Gene Amplification, and Sequencing

Total bacterial DNA was extracted from fecal samples using a QIAamp DNA Mini Kit (Qiagen, Hilden, Germany) according to the manufacturer’s protocol. The purity and concentration of the extracted genomic DNA were assessed with a NanoDrop spectrophotometer (Thermo Fisher, Waltham, MA, USA).

The library was prepared using 2 × Phanta Max Master Mix (VAZYME, China) polymerase, and the V3-V4 region of the bacterial 16S rDNA gene was amplified using the primer 338F (ACTCCTACGGGAGGCAGCA) and 806R (GGACTACHVGGGTWTCTAAT). PCR enrichment was performed in a 50 μL reaction containing a 30 ng template and fusion PCR primers. The PCR cycling conditions were as follows: 95 °C for 3 min; 30 cycles of 95 °C for 15 s, 56 °C for 15 s, 72 °C for 45 s, and final extension at 72 °C for 5 min. PCR products were purified using DNA magnetic beads (BGI, LB00V60). The validated libraries were used for sequencing on the Illumina MiSeq platform (BGI, Shenzhen, China) following the standard pipelines of Illumina and generating 2 × 300 bp paired-end reads.

To detect drug resistance genes, 10 pairs of primers were designed using Primer 5 software based on gene sequences in GenBank and prior studies [19,20,21]. The primer sequences are shown in Table S1. The PCR conditions included initial denaturation at 95 °C for 5 min, followed by 30 cycles of denaturation at 95 °C for 1 min, annealing (optimized temperature and time for specific primers), and extension at 72 °C for 1 min, with a final extension at 72 °C for 10 min. The PCR products were analyzed using 1% agarose gel electrophoresis and visualized with a gel imaging system.

### 2.3. Sequencing and Bioinformatics Analysis

The raw sequencing data underwent preprocessing to obtain clean data. Reads matching primers were processed using cutadapt v2.6 to trim contaminants, and low-quality regions were removed using a sliding window approach with a quality threshold of Q20. The overlapping paired-end reads were merged using FLASH v1.2.11, requiring a minimum overlap of 15 bp and allowing for a mismatch rate of 0.1. High-quality sequences were clustered into operational taxonomic units (OTUs) at 97% similarity using USEARCH v7.0.1090. Chimeric sequences were filtered using UCHIME v4.2.40.

### 2.4. Alpha and Beta Diversity Analysis

Rarefaction curves were generated to evaluate sequencing depth, with a cutoff of 10,000 reads per sample applied to standardize the data. Alpha diversity indices, including Chao1, ACE, Shannon, and Simpson, were calculated to evaluate within-sample diversity. The group differences in alpha diversity were visualized using boxplots and statistically compared using the Wilcoxon test. Analysis and visualization were performed using R (v3.2.1). Beta diversity analysis was conducted using QIIME (v1.80) and included Principal Coordinate Analysis (PCoA). PCoA employed an iterative algorithm to evaluate both weighted and unweighted species abundance information. The analysis utilized 75% of the sequence count from the sample with the lowest sequence depth as the subsampling threshold. Subsampling was repeated for 100 iterations, and the final statistical results and PCoA plots were generated. Beta diversity differences between groups were visualized using boxplots, generated with R (v3.4.1) and the ggplot2 package.

OTU rank curves were created to visualize species diversity across samples, and the analysis was performed using R (v3.1.1). Species accumulation curves were generated to assess the relationship between sampling effort and species richness, using R (v3.2.1). Venn diagrams were constructed to illustrate the number of shared and unique OTUs among samples, highlighting OTU overlap. The analysis was performed using R (v3.1.1) with the VennDiagram package.

### 2.5. Statistical Analysis

To analyze the differences in diarrhea rates between the suckling and weaning periods, we used a chi-square test. The test was conducted without Yates’ continuity correction to ensure robustness of the results. Alpha diversity indices were expressed as means ± standard deviation (SD). Group comparisons for alpha diversity indices and the relative abundances of the top 10 phyla and genera were performed using the independent-sample *t*-test for normally distributed data or the Mann–Whitney U-test for non-normally distributed data. Differences with a *p*-value < 0.05 were considered statistically significant, and *p* < 0.01 was considered highly significant. To compare bacterial community structures, a one-way analysis of similarity (ANOSIM) was used. LEfSe (Linear Discriminant Analysis Effect Size) was performed with a size–effect threshold of 2.0 based on LDA scores to identify differentially abundant taxa between groups.

## 3. Results

### 3.1. Diarrhea Rate

A total of 1685 calves were investigated for diarrhea, among which 153 calves experienced diarrhea, resulting in an overall diarrhea rate of 9.08%. Among the diarrheic calves, 137 cases occurred during the suckling period, accounting for 8.13% of all calves and 89.54% of the total diarrheic calves. In contrast, 16 cases occurred during the weaning period, representing 0.95% of all calves and 10.46% of the total diarrheic calves. Statistical analysis revealed that both the diarrhea rate and the proportion of total diarrheic calves were significantly higher during the suckling period compared to the post-weaning period (*p* < 0.001) (Table 1).

### 3.2. Bacterial Diversity of the Fecal Microbiota of Pre-Weaning Calves

**Figure 1:**
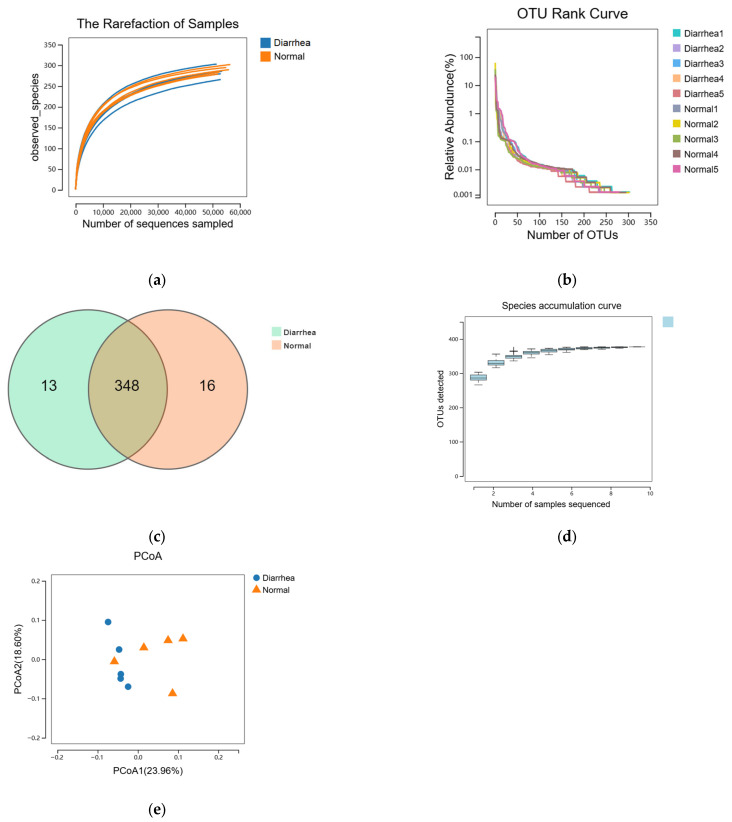
Comparative analysis of the fecal microbiome structure. The rarefaction curves (**a**) and rank abundance curves (**b**) were used to estimate the alpha diversity of the fecal microbiota in healthy and diarrheic calves. (**c**) Venn diagram illustrating the common and exclusive OTUs in the fecal microbiota of the two groups. (**d**) Species accumulation curve. The *x*-axis shows the number of samples and the *y*-axis shows the number of OTUs. The species accumulation curve reflects the influence of sampling number on species diversity. (**e**) Principal Co-ordinate Analysis based on the relative abundance of OTUs with Bray–Curtis distances, showing the differences between each individual calf. Normal, healthy calf, n = 5; diarrhea, diarrheic calf, n = 5.

To elucidate the fecal microbiota in pre-weaning calves, we performed 16S rRNA sequencing on ten fecal samples collected from the rectal–anal junction, utilizing the Illumina MiSeq platform. A total of 1,712,088 raw sequence reads were acquired five 5 healthy and five diarrheal fecal samples. After quality trimming and chimera checking, 1,568,006 clean reads remained, representing 91.58% of the valid reads. The rarefaction curves depicted in Figure 1a confirmed that the sequencing depth was adequate to encompass the overall diversity of bacteria. The Good’s coverage values exceeded 99.9% for all samples, illustrating high-quality sequencing. The rank-abundance curves demonstrated that only a limited number of bacterial species contributed to more than 1% of the overall abundance within the fecal microbiota, suggesting a community dominated by a few taxa (Figure 1b). At a sequence-similarity level of 97%, 377 operational taxonomic units (OTUs) were identified, with a mean of 287 ± 11 (range: 266–303) OTUs per sample. Venn diagram analysis revealed 348 shared OTUs between healthy and diarrheic calves, along with 13 OTUs unique to the diarrheic group (Figure 1c). These 348 shared OTUs dominated the fecal microbiota and represented 96.34% and 95.60% of the total OTU abundance in the diarrhea and healthy calves, respectively. The fecal microbiota of healthy and diarrheic calves exhibited similar microbial diversity, sharing the majority of bacterial species. The species accumulation curve for each group indicated that only a small proportion of bacteria exceeded 1% relative abundance in each sample group (Figure 1d). This observation suggests that the fecal microbiota of calves, regardless of health status, exhibits a high degree of diversity skewed toward low-abundance taxa. PCoA analysis revealed separation between samples from diarrheic and healthy calves, with one diarrheic sample clustering with the healthy group. No differences in species diversity and richness were detected among the different groups (Figure 1e).

**Figure 2:**
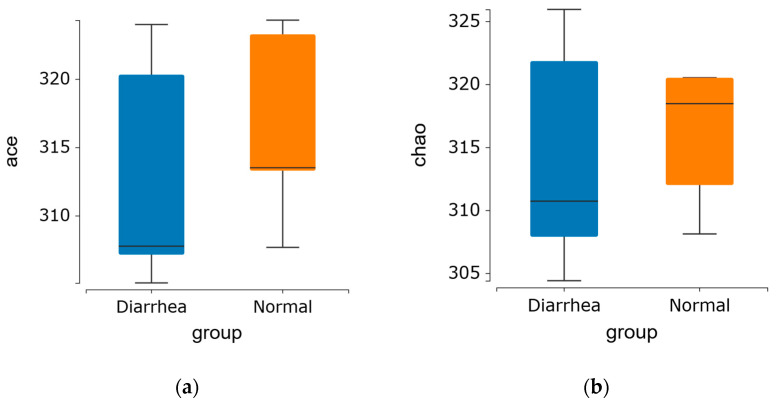

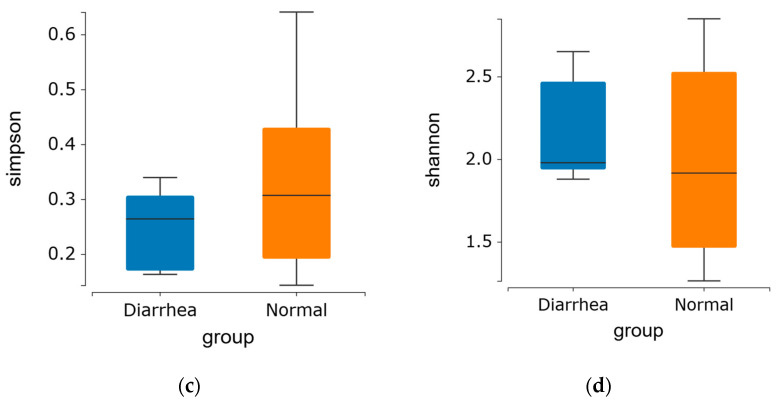
Alpha diversity index of the fecal microbiome in healthy and diarrheic calves. (**a**) Ace and (**b**) Chao1 indices represent OTU richness within the samples. (**c**) Shannon and (**d**) Simpson indices indicate the diversity of OTUs in the samples.

Alpha diversity demonstrated no significant difference in the indices among the groups, indicating that the diversity of fecal microbiota in diarrheic calves was comparable to that of healthy calves in this experiment (Figure 2a–d).

### 3.3. Diarrhea-Associated Alterations in the Fecal Microbiota

**Figure 3:**
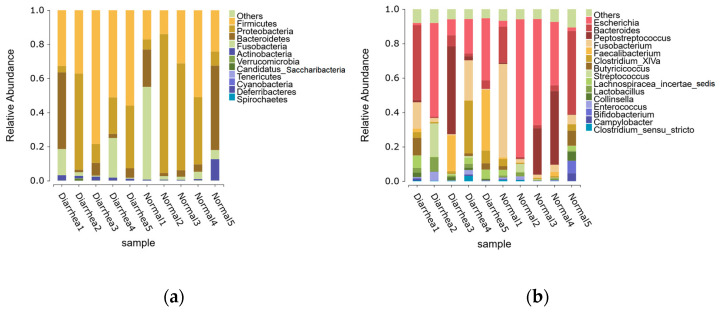
Histogram of relative abundance: (**a**) phyla with relative abundance greater than 0.5% and (**b**) genera with relative abundance greater than 0.5%. The *x*-axis shows the sample name and the *y*-axis shows the relative abundance of the species. Species with a relative abundance of less than 0.5% were combined as “Others”.

Seven distinct phyla were recognized within the fecal microbiota across all samples, with Firmicutes, Proteobacteria, Bacteroidetes, Fusobacteria, and Actinobacteria dominating. These bacteria constituted 99.78% and 99.62% of the detectable reads in the healthy and diarrhea groups, respectively (Figure 3a). Across all samples, a total of 42 genera were detected, each exhibiting an abundance of 0.5% or higher within at least one of the groups. These genera represented 99.21% and 99.39% of the overall genera abundance in the healthy and diarrheic groups, respectively. (Figure 3b).

The composition of the most abundant genera also differed between healthy and diarrheic calves. The top five genera in the healthy group were *Escherichia, Bacteroides, Peptostreptococcus, Fusobacterium*, and *Butyricicoccus*, while the diarrheic group was dominated by *Escherichia, Faecalibacterium, Bacteroides, Peptostreptococcus*, and *Clostridium_XlVa*. Genus-level analysis revealed a notable increase in *Fusobacterium, Butyricicoccus, Lachnospiracea incertae sedis, Streptococcus*, and *Lactobacillus* in the fecal microbiota of diarrheic calves compared to healthy calves. These findings suggest that certain genera, particularly those involved in fermentation and inflammation processes, may play a key role in the development of diarrhea.

**Figure 4:**
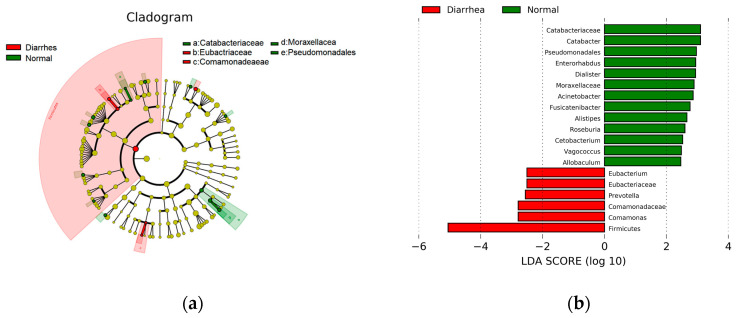
LefSe analysis. (**a**) The cladogram diagram. The same color indicates different groups, and nodes in different colors indicate microbiomes that play an important role in the group represented by the color. A color circle represents a biomarker, and the legend in the upper right corner is the biomarker’s name. The yellow nodes represent microbial groups that do not play an important role in the different groups. From the inside to the outside, each circle in turn is the phyla, class, order, family, and genus level species. (**b**) The LDA effect size plots. Green indicates healthy enriched genera and red indicates diarrhea enriched genera. Only the absolute value greater than 2 is plotted.

To identify genera linked to diarrhea, the LEfSe was utilized, focusing on the 19 most prevalent bacterial genera found in the fecal microbiota of both healthy and diarrheic calves. The cladogram (Figure 4a) depicts differences in 19 taxa between the healthy and diarrheic groups. Relative to healthy calves, *Eubacterium, Eubacteriaceae, Prevotella, Comamonadaceae, Comamonas*, and *Firmicutes* were discriminately enriched (LDA scores > 2) in diarrheic calves (Figure 4b).

### 3.4. Interactions Among Bacterial Genera in the Fecal Microbiota

**Figure 5:**
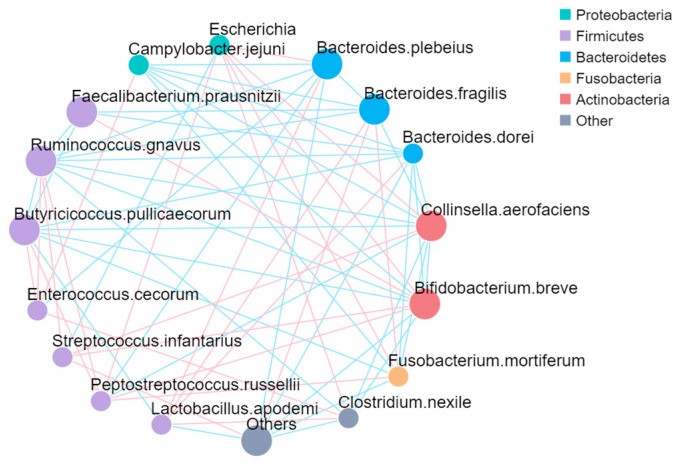
The network plot. Each node in the figure represents a species, and the color of the dots indicates that among these groups, the relative abundance of the group is the highest. The larger the area of the dots, the higher the average relative abundance of the species. Straight lines are used to connect species to species, with pink indicating a positive correlation, blue indicating a negative correlation, and the thickness of the lines indicating the size of the correlation.

We evaluated the relationships among the five most abundant bacterial phyla and sixteen species present in the fecal microbiota of both healthy and diarrheic calves (Figure 5). *Escherichia* from the phylum Proteobacteria exhibited a positive correlation with *Bacteroides. plebeius, Collinsella. aerofaciens, Bifidobacterium. breve, Fusobacterium. mortiferum, Clostridium. nexile*, and *Peptostreptococcus. russellii*. This suggests potential synergistic relationships between *Escherichia* and these genera, which may contribute to gut dysbiosis during diarrhea. In contrast, *Campylobacter. jejuni* from the Proteobacteria phylum showed a negative correlation with Bacteroidetes, Actinobacteria, and Fusobacteria. This negative correlation might indicate competitive exclusion or antagonistic interactions between Campylobacter and these bacterial groups. Bacteria from Bacteroidetes, including *Bacteroides. plebeius, Bacteroides. fragilis*, and *Bacteroides. dorei*, were negatively correlated with *Campylobacter. jejuni* and *Ruminococcus. gnavus*, but positively correlated with *Lactobacillus. apodemi*. Genera from Actinobacteria, such as *Collinsella. aerofaciens* and *Bifidobacterium. breve*, were negatively correlated with *Campylobacter. jejuni, Ruminococcus. gnavus, Butyricicoccus. pullicaecorum*, and *Clostridium. nexile* but positively correlated with *Escherichia* and *Streptococcus. infantarius*. *Lactobacillus. apodemi* showed a positive correlation with all *Bacteroidetes. Streptococcus*. *infantarius* exhibited a positive correlation with Actinobacteria, including *Collinsella. aerofaciens* and *Bifidobacterium. breve*. These interactions suggest that the Actinobacteria group may play a modulatory role in maintaining microbial balance in the gut.

### 3.5. Drug Resistance Analysis in Calves’ Fecal Samples

Table 2 presents the PCR detection outcomes and the prevalence of drug resistance genes. In all calves, the detection rates of quinolone resistance analysis genes gyrA, and gyrB were 90%, and 100%. The detection rates of β-lactam resistance analysis genes _bla_TEM, and _bla_SHV were 100%, and 10%. Chloramphenicol resistance genes floR and catA1 were detected at 100% and 50%. The detection rates of tet B and tet D, the tetracycline resistance genes, were 80% and 100%. The aminoglycoside resistance genes aadB and aadAI were detected at 0% and 100%. In diarrheal calves, the detection rate of resistant genes gyrB, blaTEM, floR, tet D, gyrA, catA1, and tet B reached 100%. In healthy calves, the detection rates of resistant genes tet B and gyrA were only 60% and 80%, respectively, and no catA1 was detected.

## 4. Discussion

Diarrhea in pre-weaning calves is a significant health challenge for the livestock industry, resulting in high rates of morbidity, mortality, and considerable economic losses. This condition is often caused by a combination of infectious agents, nutritional imbalances, and environmental stressors, which together disturb the delicate gut microbiota. The gut microbiota is essential for calf health and development, influencing nutrient absorption, immune function, and disease resistance [22,23,24,25]. In this study, we investigated diarrhea incidence, associated microbial changes, bacterial interactions, and antibiotic resistance in the fecal microbiota of both healthy and diarrheic calves. Among the 1685 calves studied, the overall diarrhea incidence was 9.08%, with 89.54% of cases occurring during the suckling period and 10.46% occurring post weaning. Despite differences in health status, the dominant bacterial phyla across the samples were Firmicutes, Proteobacteria, Bacteroidetes, Fusobacteria, and Actinobacteria, with healthy calves showing a higher relative abundance of Firmicutes, suggesting its potential role in maintaining gut health.

Our analysis of the fecal microbiota in pre-weaning calves, especially in the context of diarrhea, revealed notable differences between the healthy and diarrheic groups. Using 16S rRNA sequencing, we identified 377 operational taxonomic units (OTUs), providing a detailed perspective on bacterial diversity. Venn diagram analysis indicated that, while many OTUs were shared between the groups, 13 were unique to diarrheic calves, suggesting a distinct microbial profile linked to diarrhea. This aligns with previous research showing that gut microbiota shifts are associated with gastrointestinal disturbances, implicating specific bacterial groups in disease pathogenesis [26,27].

At the genus level, we observed a significant increase in *Fusobacterium* and *Butyricicoccus* in diarrheic calves compared to healthy ones. LEfSe analysis confirmed these results, showing that *Eubacterium* and *Prevotella* were more abundant in the diarrheic group. Elevated *Prevotella* levels have been linked to small intestinal bacterial overgrowth and diarrhea-predominant irritable bowel syndrome [28,29,30]. Additionally, correlations between *Prevotella copri* and intestinal mucosal inflammation suggest a potential role in the pathogenesis of diarrhea, possibly contributing to gut toxicity. These genera have also been associated with dysbiosis and inflammation in various animal models, reinforcing their potential role in calf diarrhea development [31,32,33].

Our findings suggest that certain bacterial interactions may play a protective role. For example, a positive correlation between *Escherichia* and beneficial genera such as *Bacteroides*, *Collinsella*, and *Bifidobacterium* suggests a symbiotic relationship beneficial for gut health. In contrast, the negative correlation between *Campylobacter jejuni* and phyla like *Bacteroidetes* and *Actinobacteria* underscores the pathogenic role of *Campylobacter*, a known gastrointestinal pathogen [34,35]. Conditional effects analysis further revealed complex interactions involving *Bacteroides* species, which showed both positive and negative correlations with other genera, indicating that while some *Bacteroides* species support gut health, others may contribute to dysbiosis, especially when pathogens are present. These insights suggest that targeting specific bacterial genera could offer promising strategies for managing calf diarrhea.

In addition to microbiota shifts, we identified high prevalence rates for several antibiotic resistance genes, including *gyrB*, *blaTEM*, *floR*, *tetD*, *gyrA*, *catA1*, and *tetB* in diarrheic calves, with significantly lower detection rates for certain genes like *tetB* and *gyrA* in healthy calves. The quinolone resistance gene *gyrA* was detected in 90% of calves overall and 100% of diarrheic calves carried *gyrB*, indicating fluoroquinolone resistance. Beta-lactam resistance genes, particularly *blaTEM*, were prevalent, while *blaSHV* was absent in diarrheic calves, which may point to potential selection pressures in different microbiota environments [17,21]. Among the beta-lactamase resistance genes, blaTEM showed the highest prevalence at 100%, followed by blaSHV at 10%, although blaSHV was absent in diarrheic calves. Enterobacteriaceae can acquire extended-spectrum beta-lactamase (ESBL) genes like blaTEM and blaSHV through mutation or horizontal gene transfer, leading to resistance to oxyimino-cephalosporins. These ESBL genes are common, with blaTEM and blaSHV being the most frequent [19,20]. Additionally, the study identified chloramphenicol resistance genes floR (100%) and catA (50%) in calf feces, with catA present in all diarrheic calves. Chloramphenicol resistance typically arises from enzymatic inactivation by chloramphenicol acetyltransferases (CATs), specifically CAT-A and CAT-B, or efflux mechanisms mediated by floR genes [18].

In conclusion, this study revealed significant differences in the gut microbiota composition of healthy and diarrheic calves, with genera such as *Eubacterium*, *Prevotella*, and *Comamonas* being more abundant in diarrheic calves. The higher prevalence of antibiotic resistance genes in diarrheic calves further highlights the potential risks associated with diarrhea-related dysbiosis. Future studies should expand on these results to explore additional factors influencing microbiota composition and disease susceptibility.

## Figures and Tables

**Table 1:** The incidence of diarrhea in calves.

Category	Total Calves	Diarrheic Calves	Diarrhea Rate *	*p*-Value	Proportion of Total Diarrheic Calves *	*p*-Value
	(n)	(n)	(%)	Weaned Period	(%)	Weaned Period
Calves	1685	153	9.08		100	
Suckling period		137	8.13	<0.001	89.54	<0.001
Weaned period		16	0.95		10.46	

* Note: Diarrhea Rate (%) represents the proportion of diarrheic calves relative to the total number of calves. Proportion of Total Diarrheic Calves (%) indicates the percentage of diarrheic calves at a specific stage (suckling or weaning) relative to the total number of diarrheic calves.

**Table 2:** Detection rate of drug resistance in calves’ fecal samples.

Group	Antibiotic	Drug Resistance Gene	Positive	Carrying Rate (%)
Calves	quinolone	gyrA	9	90
		gyrB	10	100
	β-lactam	_bla_TEM	10	100
		_bla_SHV	1	10
	chloramphenicol	floR	10	100
		catA1	5	50
	tetracycline	tet B	8	80
		tet D	10	100
	aminoglycoside	aadB	0	0
		aadAI	8	80
Healthy calves	quinolone	gyrA	4	80
	quinolone β-lactam chloramphenicol tetracycline aminoglycoside	gyrB	5	100
	β-lactam	_bla_TEM	5	100
	quinolone β-lactam chloramphenicol tetracycline aminoglycoside	_bla_SHV	1	20
	chloramphenicol	floR	5	100
	quinolone β-lactam chloramphenico tetracycline aminoglycoside	catA1	0	0
	tetracycline	tet B	3	60
	quinolon β-lactam chloramphenicol tetracycline aminoglycoside	tet D	5	100
	aminoglycoside	aadB	0	0
		aadAI	4	80
Diarrheal calves	quinolone	gyrA	5	100
		gyrB	5	100
	β-lactam	_bla_TEM	5	100
		_bla_SHV	0	0
	chloramphenicol	floR	5	100
		catA1	5	100
	tetracycline	tet B	5	100
		tet D	5	100
	aminoglycoside	aadB	0	0
		aadAI	4	80

## Data Availability

The raw data supporting the conclusions of this study will be provided to the reader upon request.

## Supplementary Materials

The following supporting information can be downloaded at: https://www.mdpi.com/article/10.3390/life15010010/s1, Table S1: The sequences of PCR primer for different drug resistance genes.
