# Supplementary material for: Gut Microbial Composition and Antibiotic Resistance Profiles in Dairy Calves with Diarrhea
Source: Life (Basel). 2024 Dec 26;15(1):10. doi: 10.3390/life15010010 (PMC11766533; doi:10.3390/life15010010)
Supplement: Supplementary file 1 [file life-15-00010-s001.zip › life-3319859-supplementary.pdf]

Gut Microbial Composition and Antibiotic Resistance Profiles in Dairy Calves with Diarrhea

Lu Zhang [1,2,3,4], Jun Bai [1,2,3], Qian Guo [5], Long Li [1,3,4], Yanqing Jia [1,2,3,4], Xinxin Qiu [1,2,3], Dong Zhou [6], Zhencang Zhang [1,2,3,*] and Huafeng Niu [1,3,*]

[1] Department of Animal Engineering, Yangling Vocational & Technical College, Yangling 712100, China; luzhang2021@nwafu.edu.cn (L.Z.); wshbj@163.com (J.B.); lilong1101@126.com (L.L.); yqjia1987@163.com (Y.J.); ylzyqiuxinxin@163.com (X.Q.)

[2] Shaanxi Engineering Research Center of the Prevention and Control for Animal Disease, Yangling Vocational & Technical College, Yangling 712100, China

[3] Key Laboratory for Efficient Ruminant Breeding Technology of Higher Education Institutions in Shanxi Province, Yangling Vocational and Technical College, Yangling 712100, China

[4] The Youth Innovation Team of Shaanxi Universities, Yangling Vocational and Technical College, Yangling 712100, China

[5] ShaanXi Province Management Station of Animal Health and Slaughter, Xi'an 710000, China; 18392957099@163.com

[6] College of Veterinary Medicine, Northwest A&F University, Yangling 712100, China; zhoudong1949@nwafu.edu.cn

* Correspondence: z13572436889@126.com (Z.Z.); nhf780206@163.com (H.N.)

Table S1 the sequences of PCR primer for different drug resistance genes

genes	primer sequences（5'-3'）	Product size (bp)	annealing temperature (℃)
gyrA	F: GGTGACGTAATCGGTAAATA<br>R: ACCATGGTGCAATGCCACCA	810	53
gyrB	F: GGACAAAGAAGGCTACAGCA<br>F: CGTCGCGTTGTACTCAGATA	879	53
$_{bla}$TEM	F: GTATCCGCTCATGAGACAATA<br>R: AGAAGTGGTCCTGCAACTTT	717	53
$_{bla}$SHV	F: ATGCGTTATATTCGCCTGTG<br>R: TTAGCGTTGCCAGTGCTCGA	860	56
floR	F: GAACACGACGCCCGCTAT<br>R: TTCCGCTTGGCCTATGAG	601	55
cat	F:AGTTGCTCAATGTACCTATAACC<br>R:TTGTAATTCATTAAGCATTCTGCC	547	55
tet B	F: TTGGTTAGGGGCAAGTTTTG<br>R: GTAATGGGCCAATAACACCG	659	58
tet D	F: AAACCATTACGGCATTCTGC<br>R: GACCGGATACACCATCCATC	787	58
aadB	F:GAGGAGTTGGACTATGGATT<br>R: CTTCATCGGCATAGTAAAA	208	53
aadAI	F: GCAGCGCAATGACATTCTTG<br>R: ATCCTCGGCGCGATTTTG	282	53
